# Correlation of pharmacodynamic activity, pharmacokinetics, and anti-product antibody responses to anti-IL-21R antibody therapeutics following IV administration to cynomolgus monkeys

**DOI:** 10.1186/1479-5876-8-41

**Published:** 2010-04-26

**Authors:** Yulia Vugmeyster, Scott Allen, Pamela Szklut, Andrea Bree, Mark Ryan, Margery Ma, Vikki Spaulding, Deborah Young, Heath Guay, Laird Bloom, Michael W Leach, Margot O'Toole, Karissa Adkins

**Affiliations:** 1 Pfizer, Inc., Andover, MA, 01810, USA

## Abstract

**Background:**

Anti-IL-21R antibodies are potential therapeutics for the treatment of autoimmune diseases. This study evaluated correlations between the pharmacodynamic (PD) activity, pharmacokinetics, and anti-product antibody responses of human anti-IL-21R antibodies Ab-01 and Ab-02 following IV administration to cynomolgus monkeys.

**Methods:**

The PD assay was based on the ability of recombinant human IL-21 (rhuIL-21) to induce expression of the IL-2RA gene in cynomolgus monkey whole blood samples *ex vivo*. Monkeys screened for responsiveness to rhuIL-21 stimulation using the PD assay, were given a single 10 mg/kg IV dosage of Ab-01, Ab-02, or a control antibody (3/group), and blood samples were evaluated for PD activity (inhibition of IL-2RA expression) for up to 148 days. Anti-IL-21R antibody concentrations and anti-product antibody responses were measured in serum using immunoassays and flow cytometry.

**Results:**

Following IV administration of Ab-01 and Ab-02 to cynomolgus monkeys, PD activity was observed as early as 5 minutes (first time point sampled). This PD activity had good correlation with the serum concentrations and anti-product antibody responses throughout the study. The mean terminal half-life (t_1/2_) was ~10.6 and 2.3 days for Ab-01 and Ab-02, respectively. PD activity was lost at ~5-13 weeks for Ab-01 and at ~2 weeks for Ab-02, when serum concentrations were relatively low. The estimated minimum concentrations needed to maintain PD activity were ~4-6 nM for Ab-01 and ~2.5 nM for Ab-02, and were consistent with the respective K_D _values for binding to human IL-21R. For Ab-01, there was noticeable inter-animal variability in t_1/2 _values (~6-14 days) and the resulting PD profiles, which correlated with the onset of anti-product antibody formation. While all three Ab-01-dosed animals were positive for anti-Ab-01 antibodies, only one monkey (with the shortest t_1/2 _and the earliest loss of PD activity) had evidence of neutralizing anti-Ab-01 antibodies. All three Ab-02-dosed monkeys developed neutralizing anti-Ab-02 antibodies.

**Conclusions:**

For anti-IL-21R antibodies Ab-01 and Ab-02, there was good correlation between PD activity and PK profiles following IV administration to cynomolgus monkeys. Compared with Ab-01, Ab-02 was eliminated markedly faster from the circulation, which correlated with a shorter duration of PD activity.

## Background

Interleukin 21 (IL-21) is a type I cytokine that is produced by activated CD4+ T cells and natural killer (NK) T cells [1-4]. IL-21 signals via the IL-21 receptor (IL-21R), which is comprised of the high affinity alpha IL-21R chain and the common gamma chain [5]. The common gamma chain is also a part of the receptor complex for other cytokines, such as interleukins 2, 4, 7, 9, and 15. Engagement of IL-21R by IL-21 leads to signaling via the Janus kinase/signal transducer and activator of transcription (JAK/STAT) pathway (reviewed in [3,4]). IL-21R is expressed by a number of cell types, including lymphoid cells (such as T, B, NK, and NKT cells), fibroblasts, keratinocytes, and intestinal epithelial cells [4,6-9]. IL-21/IL-21R signaling induces expression of multiple immune function-related genes and results in pleiotropic effects on the immune system. IL-21 promotes B cell activation and antibody production and is also an important growth factor for the TH17 lymphocyte subset, commonly associated with chronic inflammation [3,4,10,11]. IL-21 can also promote differentiation of NK cells and cells of the granulocyte and macrophage lineage, as well as enhance function of CD8+ T cells and NK T cells. Treatment of mice with an IL-21R-Fc fusion protein reduced disease markers in mouse models of systemic lupus erythematosus, rheumatoid arthritis, and inflammatory bowel disease [11-13]. Thus, selective neutralization of the IL-21/IL-21R signaling pathway is a promising approach for the treatment of a variety of autoimmune diseases.

Ab-01 and Ab-02 are human neutralizing anti-IL-21R antibodies generated by phage display technology. Ab-01 and Ab-02 bind to the same epitope on the human IL-21R, but differ in K_D _values for the human IL-21R (~2 and 0.4 nM, respectively) [14,15]. This difference in K_D _values for human IL-21R between the two human anti-IL-21R antibodies is primarily driven by the slower k_off _rate constant for Ab-02. The binding affinities of Ab-01 and Ab-02 to cynomolgus monkey IL-21R are similar to the respective values for human IL-21R. To support preclinical development of Ab-01 and Ab-02, pharmacokinetic (PK) profiles of Ab-01 and Ab-02 were evaluated in cynomolgus monkeys [14]. These initial PK studies in cynomolgus monkeys indicated that Ab-02 was cleared from the blood markedly faster compared to Ab-01 following a single IV administration. However, because of the high affinity of Ab-02 for its target and slow k_off _rate, the possibility that pharmacodynamic (PD) activity of Ab-02 persisted beyond disappearance of drug from the circulation could not be excluded.

The study presented in this manuscript was conducted to monitor the PD activity of Ab-01 and Ab-02 in cynomolgus monkeys following IV administration, and to correlate PD activity with serum concentrations of these antibodies and the presence of an anti-product antibody response. The PD assay used in this study was based on the ability of recombinant human IL-21 (rhuIL-21) to induce expression of interleukin-2 receptor alpha (IL-2RA), IL-21R, perforin (PRF1), granzyme B (GZMB), and/or interleukin 6 (IL-6) in cynomolgus monkey whole blood samples *ex vivo *(Arai *et al*, manuscript in preparation). Prior to conducting the *in vivo *study, inter-animal variability in responsiveness to *ex vivo *rhuIL-21 stimulation for these five previously identified genes in blood samples of untreated monkeys was examined to guide animal selection for this study. Monkeys pre-screened in the PD assay for responsiveness to rhuIL-21 stimulation (based on the magnitude of IL-2RA expression), were administered a single 10 mg/kg IV dosage of Ab-01, Ab-02, or a control antibody (3/group), and whole blood samples were evaluated for PD activity (i.e. inhibition of rhuIL-21-induced expression of IL-2RA). Anti-IL-21R antibody concentrations and anti-product antibody responses were measured in serum using immunoassays and flow cytometry.

## Methods

### Test Articles

Human anti-IL-21R antibodies (IgG1,λ) Ab-01 (clone VL6, also referred to as ATR-107) and Ab-02 (clone VL9), as well as a human anti-tetanus toxin IgG1 isotype control antibody were produced at Wyeth and formulated in 10 mM L-histidine, pH 6.0, containing 5% sucrose.

### Animals

For the characterization of responsiveness to *ex vivo *rhuIL-21 stimulation in the whole blood PD assay, 37 protein-naïve cynomolgus monkeys (13 males and 24 females housed at Wyeth Research, Andover MA and Pearl River, NY, respectively) were used. Nine of these monkeys (males, Andover, MA) were enrolled into the *in vivo *study, based on the magnitude of IL-2RA gene expression and their health status prior to dosing. Wyeth Institutional Animal Care and Use Committees approved all aspects of these experiments.

### *In vivo *study design

Groups of 3 male protein-naive cynomolgus monkeys were dosed with 10 mg/kg of Ab-01 (Group A), Ab-02 (Group B), or IgG control antibody (Group C). The dose was administered intravenously (infusion rate of ~4 mL/min) into the saphenous vein with a dose volume of 2.5 mL/kg.

Blood samples (~7.0 mL) for the determination of PD activity (all three groups) were collected into tubes containing sodium citrate as the anticoagulant. Blood samples (~3.0 mL) for the determination of serum Ab-01 or Ab-02 concentrations and for the evaluation of anti-product antibodies were collected into tubes without anticoagulant, allowed to clot at room temperature for approximately 15 minutes, and processed for serum by centrifugation. The sample collection schedule is shown in Table 1. Note that after day 50, additional sampling time points were added for animals 1 and 3 in the Ab-01 group (Group A) to demonstrate reversibility of PD activity, as these animals still had suppression of rhuIL-21-induced IL-2RA stimulation at day 50.

**Table 1:** *In vivo *study design and sample collection in male cynomolgus monkeys

Group (Dose) Animal #	Time points (days)	Sample collection ^a^
A; Ab-01 (10 mg/kg, IV) Animals 1-3	-13, 1(pre-^b ^and 5 min post-dose), 2, 8, 15, 22, 36, 50	Animals 1-3
	71, 92^c^	Animal 3
	92, 106, 113, 134, 148^c^	Animal 1

B; Ab-02 (10 mg/kg, IV) Animals 4-6	-13, 1(pre- and 5 min post-dose), 2, 8, 15, 22, 36	Animals 4-6

C; IgG control (10 mg/kg, IV) Animals 7-9	-13, 1(pre- and 5 min post-dose), 2, 8, 15, 22, 36	Animals 7-9

a. For Groups A and B, serum was collected to assay for test article concentrations and anti-product antibodies, and whole blood was collected for the *ex vivo *PD assay. For Group C, only whole blood samples were collected.

b. For animal 1, pre-dose day 1 samples were not collected.

c. Following PD analysis at day 50, additional sampling time points were included to demonstrate reversibility of PD activity, as these animals still had suppression of rhuIL-21 stimulation at day 50.

### *Ex vivo *whole blood assay

Whole blood samples collected from the male monkeys (Andover, MA) were placed in sterile, nuclease-free, 2 mL micro-centrifuge tubes (Axygen, Union City, CA) and treated with vehicle (10 mM L-histidine, 5% sucrose), 50 ng/mL rhuIL-21, 50 ng/mL rhuIL-21 with 30 nM IgG control antibody, or 50 ng/mL rhuIL-21 with 30 nM anti-IL-21R antibody (Ab-01 or Ab-02, as specified in Results) for 4 hrs at 37°C on a platform shaker. Whole blood samples collected from the female monkeys (Pearl River, NY) were treated with either vehicle or 20 ng/mL rhuIL-21. Peripheral blood mononuclear cells (PBMCs) in the blood samples were isolated using Ficoll methods according to manufacturer's instructions (GE Healthcare, Piscataway, NJ) and washed once in PBS.

RNA isolation was performed using the RiboPure™-Blood Kit (Applied Biosystems, Foster City, CA;, males) or RNeasy kit (Qiagen, Valencia, CA; females) according to manufacturer's instructions. RNA yield was determined using a NanoDrop 1000A spectrophotometer (NanoDrop, Wilmington, DE) and RNA quality was assessed using a 2100 Bioanalyzer (Agilent, Santa Clara, CA). RNA concentration was adjusted to 28 ng/μL (males) or 20 ng/μL (females). For RNA from the male monkeys, synthesis of cDNA was performed using a High Capacity cDNA Reverse Transcription Kit (Applied Biosystems) according to manufacturer's instructions with 700 ng of RNA, and gene expression analysis was performed using a Wyeth custom TLDA card (Applied Biosystems) designed for detection of cynomolgus monkey genes. Each cDNA synthesis reaction was mixed with TaqMan^® ^2× PCR Master Mix (Applied Biosystems), and 100 μL was loaded onto a TLDA card. TLDA cards were processed according to manufacturer instructions and amplification was performed using an ABI Prism^® ^7900HT Sequence Detection System. Cycling parameters used for each run were as follows: 50°C for 2 min, 95°C for 10 min, and 40 cycles of 95°C for 15 sec followed by 60°C for 1 min. Cycle thresholds (C_T_) were calculated using Sequence Detection Software (version 2.3, Applied Biosystems). For RNA from the female monkeys, TaqMan quantitative RT-PCR for IL-2RA only was performed using pre-qualified primers and probes to IL-2RA (Applied Biosystems; same IL-2RA primers and probes as those in custom TLDA). For both male and female monkeys, the relative quantification (RQ) of gene expression was then calculated using the delta delta Ct (ΔΔCt) method where RQ = 2^-ΔΔCt ^[16]. Zinc finger protein 592 (ZNF592, males) or protein kinase G-1 (PKG1, females) was used as the endogenous control, and the vehicle control sample was used as the calibrator for RQ calculations. Samples with RQ values greater or equal to 1.5 were considered to have gene expression higher than the corresponding vehicle control sample.

### Statistical analysis

RQ values for IL-2RA expression obtained at baseline from 37 monkeys were log-transformed and the distribution of the RQ and log [RQ] (i.e. ΔΔCt) values were tested in the Shapiro-Wilk and D'Agostino & Pearson normality tests (. The normality hypothesis was rejected for the RQ distribution (p < 0.05) but not for the log [RQ] distribution (p = 0.16 for the Shapiro Wilk test and p = 0.48 for the D'Agostino & Pearson test). The log-transformed RQ values were fitted into normal distribution (R^2 ^= 0.69).

**Figure 1:**
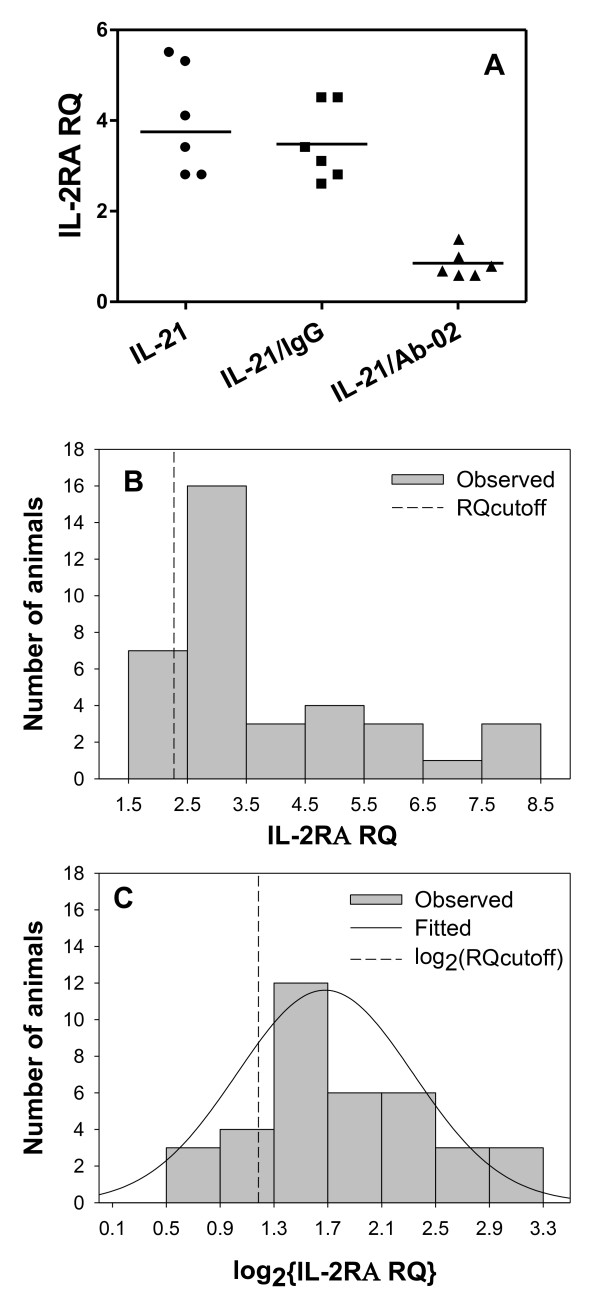
**Distribution of Relative Quantification (RQ) values for IL-2RA gene expression in whole blood of cynomolgus monkeys following *ex vivo *stimulation with rhuIL-21**. Whole blood samples were obtained from 37 protein naive cynomolgus monkeys and stimulated *ex vivo *with rhuIL-21. IL-2RA gene expression was analyzed, as described in Materials and Methods. Relative quantification (RQ) of IL-2RA gene expression was performed using a vehicle control sample, as the calibrator. To confirm that rhuIL-21-induced gene expression was dependent on engagement of cynomolgus monkey IL-21R, separate whole blood aliquots (for six monkeys) were stimulated simultaneously with either rhuIL-21 and Ab-02 (squares) or with rhuIL-21 and control IgG (triangles), and individual animal and median (solid lines) RQ values were compared with those obtained for rhuIL-21 stimulation alone (circles; A). Histogram for the RQ values (B) and log_2_-transformed RQ values (C) for 37 monkeys, as well as the fitting of these values into a Gaussian distribution (solid line) are shown. RQ_cutoff _= 2.3 and is the minimum RQ required for the enrolment into the *in vivo *study.

For comparison of the rhuIL-21-induced gene expression changes in the presence of anti-IL-21R or isotype control antibodies in untreated male monkeys (Figure 1A), Dunnett's test was performed on the log-transformed RQ values with rhuIL-21 treatment alone as the control group (p < 0.001 for rhuIL-21/Ab-02 treatment and p > 0.05 for rhuIL-21/IgG treatment).

GraphPad Prizm 5 software package (GraphPad Software Inc, San Diego, CA) was used for all statistical analyses.

### Enzyme linked immunosorbent assays (ELISA) for determination of Ab-01 and Ab-02 serum concentrations

The test articles in serum samples (in duplicate) were captured onto a microtiter plate that was coated with His_6_-tagged rhuIL-21 receptor. The bound anti-IL-21R antibody was detected with a mouse monoclonal antibody to human IgG conjugated to horseradish peroxidase.

The enzyme substrate, 3, 3', 5, 5'-tetramethylbenzidine (TMB), was used to produce a colored end-product to visualize the bound anti-IL-21R antibody. Optical densities (OD) were measured at 450 nm. Sample concentrations were determined by interpolation from a calibration curve that was fit using a 4 parameter logistic equation (Softmax Pro, version 4.3.1, Molecular Devices and Watson LIMS, version 7.0.0.01, Thermo Electron Corporation). The lower limit of quantitation (LLOQ) was 30.0 ng/mL.

### Pharmacokinetic calculations

Because of the relatively large sample volume required for the PD assay and limitations on blood volumes that could be collected from each individual cynomolgus monkey, extensive serum sampling required for determination of a complete set of PK parameters could not be performed. The only PK parameter that could be calculated under the sampling scheme employed in this study was the elimination half-life (t_1/2_). The apparent t_1/2 _was determined for each individual animal using a non-compartmental analysis module (Model 202) of the pharmacokinetic software package WinNonlin, ver. 5.1 (Pharsight, Mountain View, CA). The slope of the apparent terminal phase was estimated by log-linear regression using at least 3 data points and the terminal rate constant (λ) was derived from the slope. The apparent elimination half-life (t_1/2_) was calculated as 0.693/λ.

### Electrochemiluminescent paramagnetic bead assay for detection of anti-Ab-01 antibodies in serum

Serum samples (in duplicate) were co-incubated with biotinylated-Ab-01 and ruthenylated- Ab-01 overnight. After incubation with streptavidin-coated paramagnetic beads, the mixture and the plate were placed in the BioVeris M Series 384 Analyzer 2004 (BioVeris Corporation, Washington, DC), a magnet was applied, and unbound reactants were washed away. The emitted light was measured by photo detectors with the read out in response units (RU). Positive and negative control serum samples were included on each plate to monitor assay performance. The negative control serum samples were also used to determine the cutpoint RU, which was defined as twice the mean RU of the negative control. Samples were initially tested in a screening format at dilutions of 1:25 and 1:75. Samples generating an RU greater than or equal to the cutpoint RU were considered positive. Positive samples were reanalyzed in a full dilution series to determine the titer (the dilution that would generate an RU equal to the cutpoint RU). For positive samples, the log of the titer was reported. The minimum required dilution was 1:25 and the limit of detection was 1.40 (the log of 25). Therefore, negative samples were designated as <1.40. This assay detects both neutralizing and non-neutralizing anti-Ab-01 antibodies.

### Flow cytometry assay for detection of neutralizing anti-Ab-02 antibodies in serum

TF-1 and TF-1/huIL-21R (TF-1 cells transfected with huIL-21R; Wyeth) were grown in RPMI media containing 25 ng/ml huGMCSF (R&D Systems, Inc., Minneapolis, MN). Confluent cell cultures were centrifuged at 300 g for 10 min, resuspended in OptiMEM serum free medium (Invitrogen, Carlsbad, CA) at 10^6 ^cells/mL, and incubated at 37°C for 2 hours. The cells were then washed in cold PBS/0.5%BSA, re-suspended in ice-cold PBS buffer, and kept on ice until staining. To determine the EC_50 _for Ab-02-biotin binding to TF-1/huIL-21R cells, both the parental TF-1 and the TF-1/huIL-21R cells (10^5 ^cells per test) were incubated with either Ab-02-biotin, or IgG-biotin control using serial 3-fold dilutions (range = 16-0.0002 μg/mL) on ice for 30 minutes, washed in PBS/0.5%BSA, and then incubated with streptavidin-allophycocyanin (APC; Invitrogen). Geometric mean fluorescent intensities ("GMFI") of the APC channel peaks was collected on an LSRII flow cytometer (BD Biosciences, San Jose, CA) and analyzed using Flowjo 8.3.3 software (Tree Star, Inc., Ashland, OR). Linear regression analysis of the plots was performed using Prism 4 for Macintosh v4.0b (GraphPad Software, Inc.).

The minimum required dilution (MRD) for testing serum samples in this assay was determined to be 1:6 in PBS/0.5%BSA. To test for inhibition of Ab-02-biotin to TF-1/huIL-21R cells (i.e. for the presence of neutralizing activity), TF-1/huIL-21R cells were pre-incubated with sera from anti-IL-21R-dosed monkeys (using a 3 fold dilution series starting at the MRD), stained with an anti-IL-21R-biotin (at the estimated EC_50 _concentration), washed in PBS/0.5%BSA, stained with streptavidin-APC, and analyzed for GMFI as described above. Each serum sample was run in duplicate in two individual experiments, and the average GMFI value for the 4 replicates was obtained for each dilution point. The relative GMFI value for each serum sample for each dilution point was calculated using the formula [100%* average GMFI/average GMFI pre-dose]. A sample was considered positive if the relative GMFI value was less than or equal to 80% at the MRD. For positive samples, the log titer was calculated as the log [reciprocal dilution that would generate relative GMFI >80%]. Based on the MRD, log titers for negative samples were reported as <0.78 (log 6).

## Results

### Characterization of responsiveness of cynomolgus monkey whole blood to *ex vivo *rhuIL-21 stimulation using a PD assay

Prior to conducting the *in vivo *study, inter-animal variability in responsiveness to *ex vivo *rhuIL-21 stimulation for five previously identified genes (Arai *et al*, manuscript in preparation) in blood samples of untreated cynomolgus monkeys was examined. Gene expression changes (relative to vehicle control) for IL-2RA, IL-21R, PRF1, GZMB, and/or IL-6 following *ex vivo *stimulation of whole blood samples with rhuIL-21 for thirteen monkeys are shown in Table 2. In this assay, gene expression was quantified using relative quantification (RQ) units, as described in Materials and Methods. Not all animals had induction of gene expression of all of the above genes and there was noticeable inter-animal variability in the RQ values for all genes. Samples with RQ values greater or equal to 1.5 were considered to have gene expression higher than the corresponding vehicle control sample. IL-2RA was determined to have the largest magnitude (highest mean RQ) and most consistent change (highest percentage of animals that had RQ >1.5) in rhuIL-21-induced gene expression of the genes evaluated, and was therefore considered the best single gene for assessing PD activity of the anti-IL-21R antibodies.

**Table 2:** Relative quantification (RQ) of gene expression induced by *ex vivo *addition of rhuIL-21 to whole blood obtained from male cynomolgus monkeys

ANIMAL #	IL-2RA	IL-21R	PRF1	GZMB	IL-6
1	2.8	2.5	2.8	2.0	4.2
2	3.4	2.3	1.2	1.1	1.7
3	5.5	2.9	2.5	1.4	3.6
4	4.1	2.1	2.5	1.1	4.9
5	5.3	3.2	2.1	2.1	4.1
6	2.8	1.8	1.8	2.0	0.5
7	6.3	1.9	2.4	1.9	2.6
8	2.8	1.8	1.6	1.6	6.7
9	3.8	2.0	1.9	2.2	1.2
10	2.9	1.8	0.7	1.0	0.9
11	7.7	3.4	2.6	2.2	2.8
12	4.5	3.6	1.4	1.5	1.1
13	2.1	1.5	1.1	1.2	1.4

To confirm that rhuIL-21-induced gene expression was dependent on engagement of cynomolgus monkey IL-21R, whole blood samples from six monkeys known to be responsive to rhuIL-21 were incubated simultaneously with rhuIL-21 and the IL-21R neutralizing antibody Ab-02 (30 nM). As expected, *ex vivo *addition of Ab-02 antibody simultaneously with rhuIL-21, completely inhibited (p < 0.001) rhuIL-21-induced gene expression changes in the whole blood assay (i.e. RQ value < 1.5; Figure 1A).

To obtain a larger number of samples for characterization of the distribution of the IL-2RA response to rhuIL-21 in the *ex vivo *assay, blood samples were collected from 24 additional female cynomolgus monkeys, stimulated *ex vivo *with rhuIL-21, and analyzed for IL-2RA gene expression (in RQ units) using quantitative RT-PCR (IL-21R, PRF1, GZMB, and IL-6 expression were not analyzed for these monkeys). There were no noticeable differences in IL-2RA RQ distribution between male and female monkeys (median ± SD RQ values of 3.8 ± 1.7 and 3.0 ± 1.9, respectively), and subsequent analysis of IL-21RA RQ distribution was performed using a combined data set (n = 37). All cynomolgus monkeys tested had IL-2RA RQ values greater or equal to1.5 following *ex vivo *stimulation with rhuIL-21. The median IL-2RA RQ value (n = 37) was 3.2 with a range of 1.5 to 8.1. The distribution of the IL-2RA RQ values and log transformation (log2) of the RQ values obtained in the *ex vivo *assay are shown in Figure 1B and 1C, respectively. The distribution of IL-2RA RQ values appeared approximately lognormal, based on the normality tests described in Materials and Methods.

The minimum RQ value for IL-2RA gene expression in the *ex vivo *PD assay required for the inclusion into the *in vivo *PD study of Ab-01 and Ab-02 in cynomolgus monkeys (RQ_cutoff_) was defined as 2.3 using the formula: log [RQ_cutoff_] = mean of the log-transformed RQ values - standard deviation of the log-transformed RQ values. Approximately 81% of monkeys tested (30 of 37) had RQ values greater than 2.3 and were considered to be good responders in the *ex vivo *PD assay.

Nine male monkeys that were determined to be good responders in the *ex vivo *PD assay, were administered a single 10 mg/kg IV dosage of Ab-01, Ab-02, or a control antibody (3/group), and monitored for PD activity, serum concentration, and anti-product antibody responses at the time points shown in Table 1.

### Serum concentrations of Ab-01 and Ab-02 in cynomolgus monkeys

Initial PK studies in cynomolgus monkeys demonstrated that following single IV administration, Ab-02 was cleared markedly faster compared to Ab-01 [14]. In the PD study presented here, extensive serum sampling required for determination of a complete set of PK parameters could not be performed because of the relatively large sample volume required for the PD assay and limitations on blood volumes that could be collected from each individual cynomolgus monkey. Samples for determination of anti-IL-21R serum concentrations were taken only at those time points at which PD activity was assessed to enable correlation between the serum concentrations and PD activity for each individual animal. Thus, only elimination half-life (t_1/2_) was calculated based on the terminal phases of serum concentration-time profiles.

**Figure 2:**
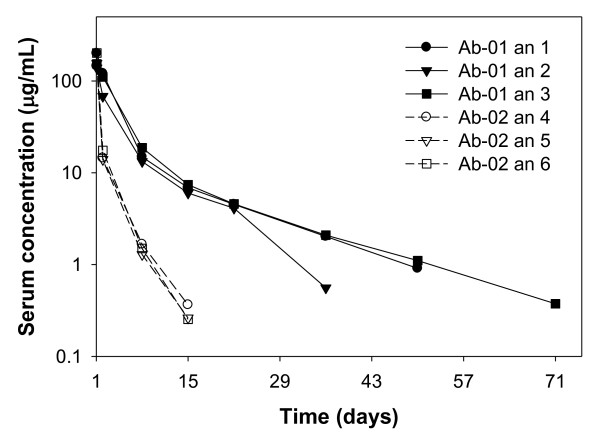
**Serum concentrations following a single 10 mg/kg IV dosage of anti-IL-21R antibody Ab-01 or Ab-02 to cynomolgus monkeys**. Serum samples were collected up to 148 days for Ab-01 (filled symbols and solid lines) and up to 36 days for Ab-02 (open symbols and dashed lines) post-dose. Ab-01 and Ab-02 serum concentrations were measured by a specific ELISA, in which his-tagged rhuIL-21 and an anti-human Fc antibody were used as a capture and a detector, respectively. Data points with serum concentrations below the LOQ of 30 ng/mL (after day 71 and day 15 for Ab-01 and Ab-02, respectively), are not shown. an = animal number.

Following a single 10 mg/kg IV dose, Ab-01 was eliminated slowly from cynomolgus monkeys, with a mean apparent terminal half-life (t_1/2_) of ~10.6 ± 3.92 days (Table 3). Up to day 22, Ab-01 serum concentrations were very similar between all three Ab-01 dosed animals (Figure 2). However, at day 36 and later time points, Ab-01 serum concentrations in animal 2 declined rapidly (to ~0.6 μg/mL) compared with those for animals 1 and 3 (to ~2 μg/mL). At day 50, animal 2 had no detectable serum Ab-01 concentration(less than LOQ of 30 ng/mL), while animals 1 and 3 had Ab-01 serum concentrations of ~0.9-1 μg/mL. Thus, the estimated t_1/2 _of Ab-01 was shorter for animal 2 (~6.2 days) compared to that for animals 1 and 3 (~12 and 14 days, respectively).

**Table 3:** Peak and last detectable concentrations, and elimination half-life after a single 10 mg/kg IV dosage of Ab-01 or Ab-02 to cynomolgus monkeys

Group	Animal	C_peak_ (μg/mL)	t_1/2_ (days)	C_last_ (μg/mL)	T_last_ (days)
A (Ab-01)	1	200	12	0.91	50
	2	139	6.2	0.56	36
	3	153	14	0.37	71
	Mean	164	11	0.61	52
	SD	32	3.9	0.27	18

B (Ab-02)	4	145	2.5	0.36	15
	5	155	2.3	0.26	15
	6	201	2.1	0.25	15
	Mean	167	2.3	0.29	15
	SD	30	0.16	0.06	0

C_peak _= Concentration at 5 minutes, the first sampling time point after IV administration; t_1/2 _= elimination half-life; T_last _= last time point at which the test article concentration was above the LOQ (30.0 ng/mL); C_last _= concentration at T_last_.

**Figure 3:**
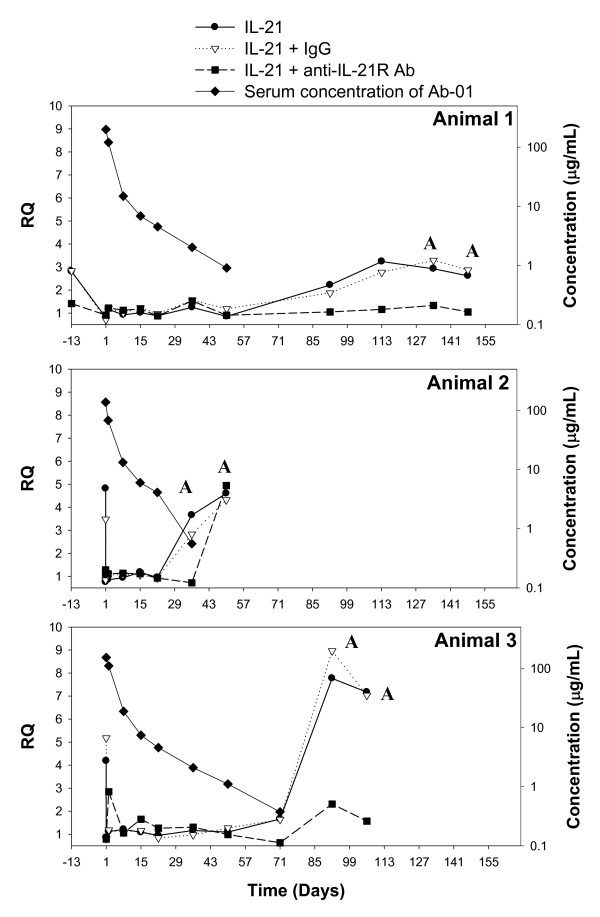
**Correlation of serum concentrations, PD activity, and anti-product antibody responses following a single 10 mg/kg IV dosage of anti-IL-21R antibody Ab-01 to cynomolgus monkeys**. Each pre-dose (day -13 for animal 1 and day 1 for animals 2 and 3) and post-dose whole blood sample was divided into four aliquots. The first and second aliquots were treated with either rhuIL-21 (filled circle) or vehicle (a calibrator for RQ calculations), respectively, and were used to assess whether circulating test article affected *ex vivo *rhuIL-21-induced IL-2RA gene expression (i.e. PD activity). The third aliquot (filled square) was treated with rhuIL-21 and Ab-01, except for day -13 samples for which Ab-02 was used. The fourth aliquot (open triangle) was treated with rhuIL-21 and an IgG control antibody (negative control for the third treatment). Ab-01 serum concentrations (filled diamonds) were measured by a specific ELISA up to days 148, 50, and 92 for animals 1, 2, and 3, respectively; data points with serum concentrations below the LOQ (30 ng/mL) are not shown. Anti-Ab-01 antibodies (neutralizing and non-neutralizing) were assessed by bead-based immunoassay; positive result indicated by "A".

**Figure 4:**
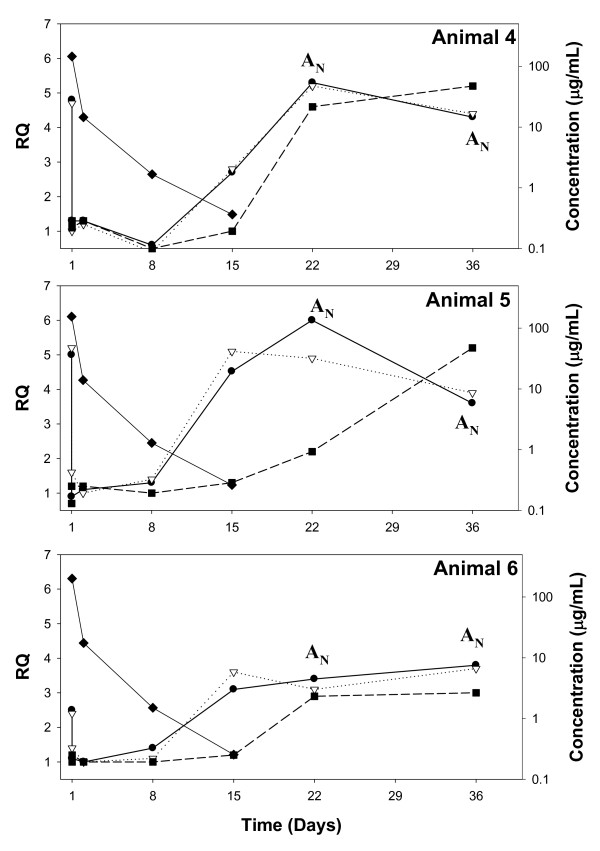
**Correlation of serum concentrations, PD activity, and anti-product antibody responses following a single 10 mg/kg IV dosage of anti-IL-21R antibody Ab-02 to cynomolgus monkeys**. Each pre-dose (day 1) and post-dose whole blood sample was divided into four 1.5 mL aliquots. First and second aliquots were treated with either rhuIL-21 (filled circle) or vehicle (a calibrator for RQ calculations), respectively, and were used to assess whether circulating test article affected *ex vivo *rhuIL-21-induced IL-2RA gene expression (i.e. PD activity). The third aliquot (filled square) was treated with rhuIL-21 and Ab-02 and the fourth aliquot (open triangle) was treated with rhuIL-21 and an IgG control antibody (negative control for the third treatment). Ab-02 serum concentrations (filled diamonds) were measured by a specific ELISA up to day 36. Data points with serum concentrations below the LOQ (30 ng/mL) are not shown. Neutralizing anti-Ab-02 antibodies were assessed by flow cytometry; positive result indicated by "A_N_".

As expected based on the initial PK studies [14], after a single 10 mg/kg IV dose, the serum concentrations of Ab-02 declined much faster than those of Ab-01 (Figure 2). Note that test article concentrations in sera were measured at all the time points at which PD activity was assessed (as shown in Table 1). However, the data points with serum concentrations below the LOQ (30 ng/mL) are not shown in Figures 2, 3 and 4. All three Ab-02-dosed monkeys had similar concentration time-profiles and apparent t_1/2 _values (Table 3), with serum concentrations declining to relatively low levels at day 15 (<0.4 μg/mL) and to less than the LOQ at day 22. The estimated mean t_1/2 _of Ab-02 was 2.3 ± 0.16 days. Ab-01 and Ab-02 concentrations started to diverge as early as 24 hrs post-dose and Ab-02 concentrations were more than 10-fold lower than Ab-01 concentrations at the one week time point. These data confirmed observations from the earlier PK studies [14], in which Ab-02 had ~5-7 fold faster CL, compared to Ab-01 (i.e. 500-700% of the Ab-01 value).

### Pharmacodynamic response

For all nine monkeys enrolled into this study, each pre-dose and post-dose blood sample was divided into four 1.5 mL aliquots. The first and second aliquots were treated with either rhuIL-21 or vehicle (a calibrator for RQ calculations), and were used to assess whether circulating test article affected *ex vivo *rhuIL-21-induced IL-2RA gene expression (i.e. demonstrated PD activity). The third aliquot was treated with rhuIL-21 and an anti-IL-21R antibody (30 nM), and the fourth aliquot was treated with rhuIL-21 and an IgG control antibody (negative control for the anti-IL-21R antibody). The third and fourth aliquots were used to assess whether inhibition of rhuIL-21-induced IL-2RA gene expression by circulating test article in a given post-dose sample was complete (for time points at which PD activity was observed), to assess whether the return of rhuIL-21-induced gene expression was mediated through the IL-21R (for time points at which PD activity was lost), and to monitor for the presence of neutralizing anti-product antibodies.

For Ab-01, complete inhibition of rhuIL-21-induced IL-2RA gene expression (IL-2RA RQ <1.5) persisted until at least day 22 for animal 2 and at least day 50 for animals 1 and 3, when serum Ab-01 concentrations were at or above 0.9 μg/mL (6 nM) for all three monkeys (Table 3 and Figure 3, data points with serum concentrations below the LOQ of 30 ng/mL are not shown). *Ex vivo *rhuIL-21-induced IL-2RA expression returned to pre-dose values (i.e. PD activity was lost) at day 92 for animals 1 and 3, coincident with the time points at which serum concentrations of the test articles were <LOQ (Figure 3). For animal 2, PD activity was lost at day 36, when serum Ab-01 concentration had declined to a relatively low level of ~4 nM (0.6 μg/mL). For all time points examined in this study, the PD activity of Ab-01 appeared to be all or none, such that there was typically either complete inhibition of rhuIL-21-induced IL-2RA gene expression (RQ < 1.5), or a lack of inhibition (RQ similar to that in the corresponding pre-dose sample). A partial PD response was difficult to differentiate because of the intra-animal variability observed in IL-2RA RQ values. It is possible that data points with partial PD response for Ab-01 would have been observed if additional sampling time points were collected. The minimum concentration that was needed to maintain minimum PD activity of Ab-01 (C_min_) could not be precisely estimated, but is likely to be ~4-6 nM.

For Ab-02, PD activity persisted until at least day 8 (RQ < 1.5), when serum Ab-02 concentrations were at or above 1.3 μg/mL (Figure 4 and Table 3). PD activity was lost (RQ > 1.5) at day 15 for all three monkeys. In blood samples obtained on day 15 from animals 5 and 6, IL-2RA RQ values appeared similar to the corresponding pre-dose values (i.e. complete loss of PD activity) and serum Ab-02 concentrations were less or equal to 0.26 μg/mL (1.7 nM). There was an apparent partial PD response in blood samples obtained on day 15 from animal 4, as the observed IL-2RA RQ value of 2.7 was less than that at pre-dose (RQ = 4.8) and at the subsequent day 22 time-point (RQ = 5.3; Figure 4). Animal 4 also had a slightly longer estimated t_1/2 _and somewhat higher Ab-02 serum concentration at day 15 (~2.5 nM), compared to animals 5 and 6. These data suggested that the C_min _of Ab-02 that was needed to maintain PD activity was approximately 2.5 nM.

For the isotype control group, *ex vivo *added rhuIL-21 induced IL-2RA gene expression in whole blood samples from all three monkeys at all time points, with noticeable intra-animal variability in the IL-2RA RQ values (data not shown).

### Anti-product antibody response

At the first time point where loss of PD activity was observed, *ex vivo *addition of an anti-IL-21R antibody simultaneous with rhuIL-21 inhibited the induction of IL-2RA gene expression (RQ < 1.5) in all Ab-01- and Ab-02-dosed monkeys, indicating that the return of rhuIL-21-induced gene expression was mediated through the IL-21R and that neutralizing anti-IL-21R antibodies were not present (Figure 3 and 4). *Ex vivo *addition of Ab-01 continued to demonstrate inhibitory activity at subsequent time points collected from animals 1 and 3. However, Ab-01 had no *ex vivo *inhibitory activity at the day 50 time point from animal 2 (Figure 3). Similarly, Ab-02 had no *ex vivo *inhibitory activity at the day 22 and/or day 36 time points collected from all animals in the Ab-02 dosed group (Figure 4). These data suggested that animal 2 in the Ab-01 group and all three animals (4-6) in the Ab-02 group had developed neutralizing anti-product antibodies.

The presence of neutralizing anti-Ab-02 antibodies in Ab-02-dosed animals was confirmed using an orthogonal flow cytometric (FACS)-based assay. In this assay, TF-1 cells transfected with human IL-21R (TF-1/hIL-21R) were stained with Ab-02-biotin in the presence of serum samples obtained from Ab-02-dosed monkeys. Streptavidin-APC was added to detect Ab-02-biotin binding to TF-1/hIL-21R cells. All three Ab-02 dosed animals tested positive in the FACS-based neutralizing antibody assay at day 22 and day 36, with log titers ranging from 2.2 to 4.1 (Table 4).

**Table 4:** Formation of neutralizing anti-Ab-02 antibodies (log Titer) after a single 10 mg/kg IV dosage of Ab-02 to cynomolgus monkeys

TIME (DAYS)	ANIMAL 4	ANIMAL 5	ANIMAL 6
**Pre-dose**	Negative	Negative	Negative
			
**15**	Negative	Negative	Negative
**22**	4.12	2.7	2.2
**36**	2.2	2.7	2.7

Formation of neutralizing anti-Ab-02 was assessed using a flow cytometric assay, in which serum samples from Ab-02 dosed monkeys were tested for inhibition (relative to pre-dose) of anti-IL-21R-biotin binding to TF-1 cells transfected with human IL-21R. Negative samples had no inhibition at the minimum required dilution of 1:6 and had a log titer < 0.78.

Since only one of the Ab-01-dosed animals showed evidence of neutralizing anti-Ab-01 antibodies in the *ex vivo *IL-2RA gene expression assay, serum samples from Ab-01-dosed monkeys were tested in an electrochemiluminescent paramagnetic bead-based assay that detected both neutralizing and non-neutralizing anti-Ab-01 antibodies. In this assay, serum samples were co-incubated with biotinylated- Ab-01 and ruthenylated- Ab-01, streptavidin coated paramagnetic beads were added to the mixture, and the emitted light was detected using BioVeris technology. All three Ab-01 dosed monkeys were positive for anti-Ab-01 antibodies in this assay, with log titers ranging from 1.86 to 3.43 (Table 5). There was significant inter-animal variability in the apparent onset of anti-Ab-01 generation. The first serum sample that was positive for anti-Ab-01 antibodies in the BioVeris-based assay was obtained at day 134, 36, and 92 for animals 1, 2, and 3 respectively. Thus, among the three Ab-01-dosed animals, animal 2 had the shortest t_1/2 _and the fastest onset and the highest titer of anti-Ab-01 antibody response. Animal 2 was also the only Ab-01-dosed monkey that showed evidence of neutralizing anti-Ab-01 antibody response in the *ex vivo *IL-2RA gene expression assay, similar to all three Ab-02 dosed monkeys.

**Table 5:** Formation of anti-Ab-01 antibodies (log Titer) after a single 10 mg/kg IV dosage of Ab-01 to male cynomolgus monkeys

TIME (DAYS)	ANIMAL 1	ANIMAL 2	ANIMAL 3
**Pre-dose**	Negative	Negative	Negative
			
**15**	Negative	Negative	Negative
**22**	Negative	Negative	Negative
**36**	Negative	2.13	Negative
**50**	Negative	3.43	Negative
**71**	ND	ND	Negative
**92**	Negative	ND	2.27
**106**	ND	ND	2.79
**113**	Negative	ND	ND
**134**	1.86	ND	ND
**148**	2.4	ND	ND

"ND" = Not determined

Formation of anti-Ab-01 antibodies (neutralizing and non-neutralizing) was assessed using an electrochemiluminescent paramagnetic bead assay, in which serum samples from Ab-01-dosed monkeys were tested for binding to biotinylated- Ab-01 and ruthenylated- Ab-01. Negative samples had no binding at the minimum required dilution of 1:25 and had a log titer < 1.40.

## Discussion

The study presented in this manuscript was conducted to monitor the PD activity of anti-IL-21R antibodies Ab-01 and Ab-02 in cynomolgus monkeys following IV administration and to correlate PD activity with serum concentrations of these antibodies and the presence of an anti-product antibody response. Since Ab-02 had slower k_off _rate leading to a lower K_D _value for *in vitro *binding to human IL-21R (~0.4 nM) compared to Ab-01 (~2 nM) [14,15]; this study also explored whether improvement in target binding affinity *in vitro *translated into an improved PK-PD profile in primates for Ab-02.

Prior to conducting the *in vivo *PD study of anti-IL-21R antibodies in cynomolgus monkeys, a single gene, IL-2RA, was identified for assessing PD activity, and inter-animal variability in IL-2RA gene expression was characterized in monkeys. The distribution of IL-2RA RQ values in response to rhuIL-21 stimulation appeared approximately lognormal, which was similar to the distribution of RQ values for genes upregulated in disease conditions or to the distribution of ΔCt values of immune response genes in blood obtained from healthy human donors [17,18]. Based on the statistical analysis of the IL-2RA RQ values in the 37 monkeys tested, the good responders to rhuIL-21 were defined as those with the RQ values ≥ 2.3 and the frequency of good responders was estimated to be ~80%. Animals that were good responders prior to the *in vivo *study also were good responders at subsequent time points collected prior to dosing. These data illustrate that for preclinical or clinical studies that rely on *ex vivo *assays for assessing PD activity, potential heterogeneity in the *ex vivo *response needs to be examined, characterized, and taken into account during study design and animal or patient selection.

Following IV administration of anti-IL-21R antibodies Ab-01 and Ab-02 to cynomolgus monkeys, there was a good correlation between the PD activity (based on inhibition of IL-2RA gene expression induced by *ex vivo *addition of rhuIL-21 to whole blood), serum concentrations, and anti-product antibody responses. Complete inhibition of rhuIL-21-induced IL-2RA gene expression (PD activity) was observed at the first time point, 5 minutes, after dosing, for both Ab-01 and Ab-02. PD activity was lost (i.e. rhuIL-21 induction of IL-2RA expression was again observed) with the elimination of Ab-01 and Ab-02 from circulation. In agreement with earlier PK studies [14], Ab-02 had faster elimination in monkeys compared with Ab-01, with a mean apparent t_1/2 _of 10.6 and 2.3 days for Ab-01 and Ab-02, respectively. At the day 15 time point, PD activity was completely or partially lost in all three Ab-02-dosed monkeys, while all three monkeys in the Ab-01 dose group had relatively high serum Ab-01 concentrations (~6.0-7.4 μg/mL) and full PD activity. Thus, Ab-01 had a longer duration of PD activity and a longer t_1/2 _in cynomolgus monkeys.

All three Ab-01-dosed monkeys were positive for anti-product antibodies, based on an assay that detected both neutralizing and non-neutralizing anti-Ab-01 antibodies. However, only one of three Ab-01-dosed animals had evidence of neutralizing anti-Ab-01 antibodies in the IL-2RA gene expression assay. There was significant inter-animal variability in the apparent terminal serum half-life of Ab-01 (~6 to 14 days), which appeared to correlate with the onset and titer of anti-product antibody response for the three Ab-01-dosed animals. Animal 2 had the shortest t_1/2_, the fastest onset, and highest titer of anti-Ab-01 antibody response. Animal 2 was the only Ab-01-dosed monkey that showed evidence of neutralizing anti-Ab-01 response in the *ex vivo *IL-2RA gene expression assay, similar to all three Ab-02-dosed monkeys. Animals 1 and 3 had noticeably longer t_1/2 _and later onset of anti-Ab-01 antibody responses.

Despite the variability in serum t_1/2 _of Ab-01 between the three Ab-01-dosed animals, there was a good correlation between Ab-01 serum concentrations and PD activity, such that PD activity was observed in all three Ab-01-dosed monkeys when serum concentrations of Ab-01 were at or above 0.9 μg/mL (~6 nM) and was lost in all three monkeys when serum concentrations were < 0.6 ng/mL (~4 nM). The minimum serum concentration of Ab-01 which was needed to maintain PD activity (C_min_) could not be precisely determined from the data obtained in this study, because Ab-01 PD activity appeared to be all or none at the time points collected and there were not enough time points taken in the lower concentration range to fully evaluate the concentration-effect relationship. However, the available concentration-effect data discussed above suggested that the C_min _of Ab-01 was ~4-6 nM.

All three of the Ab-02-dosed monkeys had similar Ab-02 concentration-time profiles, onset of anti-product antibody responses, and resulting PD-time profiles. All three Ab-02-dosed monkeys were positive for neutralizing antibodies in the two orthogonal assays (gene expression and flow cytometry) at and after day 22. In blood samples from two Ab-02-dosed monkeys, Ab-02 PD activity was all or none at time points tested. One Ab-02-dosed animal had apparent partial PD activity at one time point (day 15), when serum Ab-02 concentrations were ~2.5 nM. Based on these data, C_min _needed to maintain PD activity of Ab-02 was assumed to be approximately 2.5 nM. Additional studies would be needed to confirm the C_min _for both Ab-01 and Ab-02. However, the preliminary C_min _estimates of ~4-6 nM for Ab-01 and ~2.5 nM for Ab-02 were consistent with the K_D _values for Ab-01 and Ab-02 binding to human IL-21R (~2.0 and 0.5 nM, respectively). Thus, Ab-02 had higher binding affinity for human IL-21R and a lower estimated C_min _needed to maintain PD activity, compared to Ab-01. However, because of the fast elimination of Ab-02 from the circulation, loss of PD activity occurred much faster in Ab-02-dosed monkeys (day 15-22), compared to Ab-01 (not earlier than day 36).

The mechanism of fast elimination of Ab-02 in monkeys is not known but is unlikely to be entirely target (IL-21R)-mediated because it appeared non-saturable at a relatively high dose level (100 mg/kg) [14]. While the dosage that is needed to saturate IL-21R binding sites in cynomolgus monkeys or other species has not been defined, it is very unlikely to exceed 100 mg/kg, based on reports on other human antibodies directed against highly-expressed cell surface receptors, including an antibody to another type I cytokine receptor, IL-2R [19]. A single 2 mg/kg dosage of anti-IL-2R antibody to human subjects resulted in complete saturation of IL-2R on peripheral blood lymphocytes for ~45 days post dose, as long as the serum anti-IL-2R Ab concentration were above 1 μg/mL (~6 nM), which was similar to the C_min _needed to maintain PD activity of the anti-IL-21R antibodies Ab-01 and Ab-02 in the *ex vivo *whole blood assay following a single 10 mg/kg aose to cynomolgus monkeys. Differences in Ab-01 and Ab-02 serum concentrations observed in cynomolgus monkeys in this study and earlier PK studies are not likely to be entirely explained by neutralizing anti-product responses, because Ab-01 and Ab-02 serum concentrations started to diverge as early as 24 hrs post-dose and Ab-02 concentrations were more than 10-fold lower than Ab-01 concentrations at the one week time point. Although total body clearance (CL) of Ab-01 and Ab-02 could not be accurately estimated in this study, the observed serum concentration profiles were consistent with the previously reported faster CL of Ab-01 (~5-7 mL/hr/kg) compared to that of Ab-02 (~1 mL/hr/kg) [14]. Further studies are needed to delineate the mechanism of fast clearance of Ab-02 in monkeys.

Finally, data presented in this report, suggested that for anti-IL-21R antibody Ab-02, a lower K_D _value for target (IL-21R) binding in the *in vitro *assay (~0.4 nM for Ab-02 versus ~2 nM for Ab-01; [15,16]) did not translate into an improved PK-PD profile in primates, primarily due to differences in pharmacokinetics between the two antibodies. Thus, optimization of candidate anti-IL-21R antibodies in *in vitro *systems may not be sufficient for generation of therapeutic antibodies with improved PK-PD profiles, and PK-PD studies in non-human primates are recommended prior to first in human studies.

## Conclusions

Following IV administration of anti-IL-21R antibodies Ab-01 and Ab-02 to cynomolgus monkeys, there was good correlation between the PD activity (based on IL-2RA gene expression in *ex vivo *rhuIL-21-stimulated whole blood), the respective serum concentration profiles, and anti-product antibody responses. Compared with Ab-01, Ab-02 was eliminated markedly faster from the circulation (shorter t_1/2 _and faster clearance), which correlated with a shorter duration of PD activity. Thus, slower k_off _rate leading to a lower K_D _value for *in vitro *binding to human IL-21R of Ab-02 (~0.4 nM) compared to Ab-01 (~2 nM) did not translate into an improved PK-PD profile in primates, primarily due to differences in pharmacokinetics between the two antibodies. This study exemplifies that detailed *in vivo *PK-PD studies in non-human primates (such as those presented in this report) are crucial for the selection of lead biotherapeutic candidates for first-in-human clinical studies.

## Competing interests

All authors are current or former employees and/or hold stocks or stock options of Wyeth, Inc (currently Pfizer) at the time the manuscript was prepared.

## Authors' contributions

YV and KA drafted the manuscript. YV, KA, LB, HG, MO, AB, and DY carried out study design, conduct and data interpretatin. ML critically reviewed the manuscript and data interpretation. MO identified the biomarkers and devised the PD assay strategy for this study. PS performed immunoassay analysis. SA, MM, VS, performed gene expression analysis, and MR performed FACS analysis. All authors have read and approved the final manuscript.
